# The Effect of Prenatal Food Restriction on Brain Proteome in Appropriately Grown and Growth Restricted Male Wistar Rats

**DOI:** 10.3389/fnins.2021.665354

**Published:** 2021-04-14

**Authors:** Anastasios Potiris, Antigoni Manousopoulou, Andreas Zouridis, Polyxeni-Maria Sarli, Panagiota Pervanidou, George Eliades, Despina N. Perrea, Efthymios Deligeoroglou, Spiros D. Garbis, Makarios Eleftheriades

**Affiliations:** ^1^Second Department of Obstetrics and Gynaecology, Medical School, National and Kapodistrian University of Athens, Athens, Greece; ^2^Beckman Research Institute, City of Hope National Medical Center, Duarte, CA, United States; ^3^First Department of Paediatrics, Medical School, National and Kapodistrian University of Athens, Athens, Greece; ^4^Biomaterials Laboratory, School of Dentistry, National and Kapodistrian University of Athens, Athens, Greece; ^5^Laboratory of Experimental Surgery and Surgical Research “N.S. Christeas”, Medical School, National and Kapodistrian University of Athens, Athens, Greece; ^6^Cancer Sciences Unit, Faculty of Medicine, University of Southampton, Southampton, United Kingdom

**Keywords:** proteomics, fetal growth restriction (FGR), in utero food restriction, brain, offspring, LC-MS, fetal programming, IUGR

## Abstract

**Background:**

Fetal growth restriction (FGR) has been associated with a higher risk of developing adverse perinatal outcomes and distinct neurodevelopmental and neurobehavioral disorders. The aim of the present study was to investigate the impact of prenatal food restriction on the brain proteome in both FGR and appropriately grown rats and to identify potential pathways connecting maternal malnutrition with altered brain development.

**Methods:**

Ten time-dated pregnant Wistar rats were housed individually at their 12th day of gestation. On the 15th day of gestation, the rats were randomly divided into two groups, namely the food restricted one (*n* = 6) and the control group (*n* = 4). From days 15 to 21 the control group had unlimited access to food and the food restricted group was given half the amount of food that was on average consumed by the control group, based on measurements taken place the day before. On the 21st day of gestation, all rats delivered spontaneously and after birth all newborn pups of the food restricted group were weighed and matched as appropriately grown (non-FGR) or growth restricted (FGR) and brain tissues were immediately collected. A multiplex experiment was performed analyzing brain tissues from 4 FGR, 4 non-FGR, and 3 control male offspring. Differentially expressed proteins (DEPs) were subjected to bioinformatics analysis in order to identify over-represented processes.

**Results:**

Proteomic analysis resulted in the profiling of 3,964 proteins. Gene ontology analysis of the common DEPs using DAVID (https://david.ncifcrf.gov/) showed significant enrichment for terms related to cellular morphology, learning, memory and positive regulation of NF-kappaB signaling. Ingenuity Pathway Analysis showed significant induction of inflammation in FGR pups, whereas significant induction of cell migration and cell spreading were observed in non-FGR pups.

**Conclusion:**

This study demonstrated that in both FGR and non-FGR neonates, a range of adaptive neurodevelopmental processes takes place, which may result in altered cellular morphology, chronic stress, poor memory and learning outcomes. Furthermore, this study highlighted that not only FGR, but also appropriately grown pups, which have been exposed to prenatal food deprivation may be at increased risk for impaired cognitive and developmental outcomes.

## Introduction

Fetal growth restriction (FGR) also known as intrauterine growth restriction (IUGR), refers to newborns failing to reach their genetically predetermined growth potential (Battaglia and Lubchenco, 1967; Beune et al., 2018; ACOG, 2019). Maternal malnutrition with low-caloric diet and low maternal body weight have been associated with FGR (Wollmann, 1998; Eleftheriades et al., 2016). Experimental studies in food restricted rats have produced similar results in the rat offspring (Fowden et al., 2005; Hanson and Gluckman, 2008; Aravidou et al., 2016).

The growth restricted fetus is at higher risk of developing not only adverse perinatal outcomes, such as prematurity, stillbirth, neonatal mortality and morbidity (Figueras et al., 2007; Xu et al., 2010) but also disease in adulthood. According to the “thrifty phenotype” hypothesis, when a mother is nutritionally restricted, then the *in utero* modifications that secure fetal energy sufficiency are in priority. Even though these adaptive changes have a major impact for short-term survival of the fetus, they tend to make it more susceptible to disease in the future (Hales and Barker, 2001). Moreover, the recently described predictive adaptive responses (PAR) hypothesis (Gluckman et al., 2005) has suggested that a poor *in utero* environment induces metabolic and behavioral changes that maximize survival but reduces fitness if environment later improves. Evidence shows that FGR has been associated with obesity and the metabolic syndrome and increases the risk for diabetes type-2 and cardiovascular disease later in life (Pervanidou et al., 2006; Pedroso et al., 2017). Eventually, these FGR-evoked alterations affect both males and females, with females favoring an earlier obesity development in comparison with males (Pedroso et al., 2019).

FGR has been associated with distinct neurodevelopmental (such as autism and Attention Deficit Hyperactivity Disorder—ADHD) and neurobehavioral (such as anxiety and depression) disorders (Walker and Marlow, 2008; Lampi et al., 2012; Korzeniewski et al., 2017). Animal studies have shown that prenatal moderate food restriction alters growth and neurodevelopment in the offspring, as evidenced by behaviors indicative of impaired coordination, anxiety and impaired cognitive function (Akitake et al., 2015).

Quantitative proteomics can provide an unbiased phenotypic insight on the systemic effects of an intervention to an organ of interest. The aim of the present study was to investigate the impact of maternal food restriction on the global brain proteomic profile of appropriately grown and FGR Wistar rat offspring and to identify potential pathways connecting maternal malnutrition with altered brain development.

## Materials and Methods

This study received ethics approval by the Ethics Committee of Aretaieion University Hospital, Medical School of the National and Kapodistrian University of Athens with registration number B-207/13-10-2016. Research license and approval for experimental animal (RjHan:WI–Wistar rats) utilization was granted by the Division of Agriculture and Veterinary Policy, District of Attica, Greece (Decision 5035/21-09-2017 and its modification 1211/19-03-2018). Animal handling was performed in accordance with the local applied laws (1197/1981 and 2015/1992) for the protection of animals and the Directive 2010/63/EU of the European Parliament and Council regarding the protection of animals used for research purposes.

### Animal Model

**FIGURE 1:**
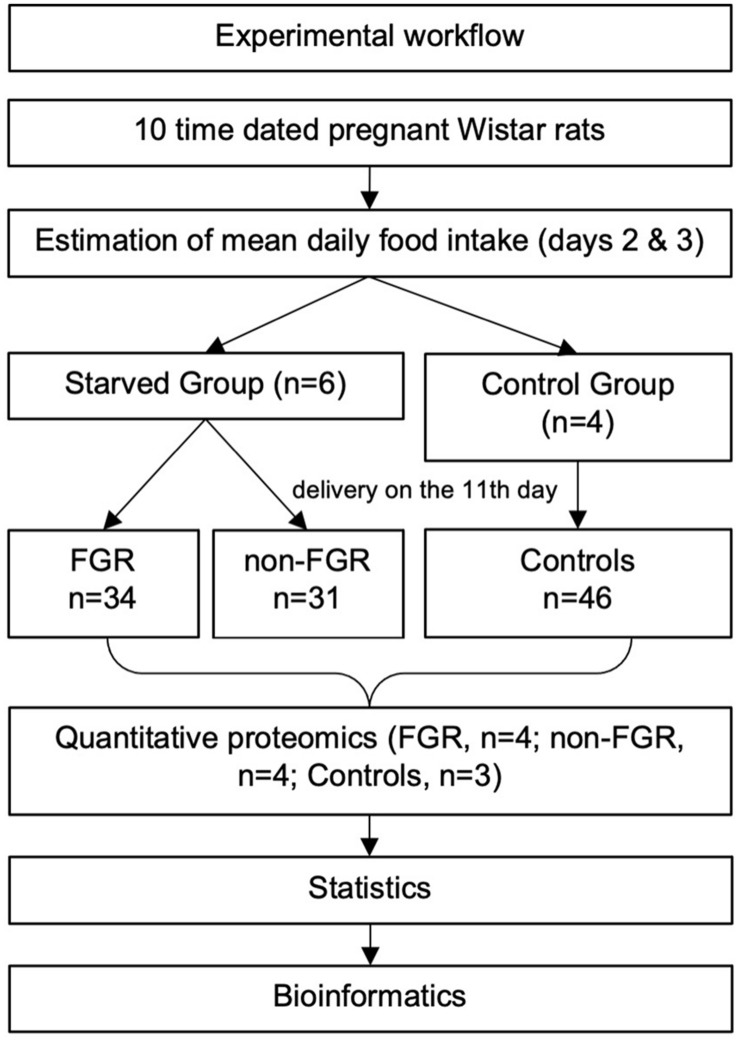
An overview of the experimental workflow.

An overview of the experimental workflow is illustrated in Figure 1. Ten time-dated pregnant Wistar rats (Janvier Labs—Rodent research models and associated services, France) at their 12th day of gestation were housed individually in the Experimental Surgery Laboratory of Aretaieion University Hospital. All rats conceived on the 22nd of March 2018 and we received them to our facilities on April 4th along with all the necessary certificates. During the whole experimental process, temperature was maintained between 22 and 23°C, humidity ranging 55–65% and 12-h light/dark cycles were applied. All animals were fed with a standardized diet 4RF21-GLP (Mucedola S.r.l., Settimo Milanese, Italy) consisting of 18.5% protein and 2.688 kCal/kg metabolizable energy. Day 1 (12th day of gestation) was considered an adjustment day, all rats were weighed, and the food consumed was not recorded. During days 2 and 3 the food consumption was systematically recorded, in order to estimate the mean daily food consumption. On day 4 (15th day of gestation) the rats were randomly divided into two groups; the food restricted one (*n* = 6) and the control group (*n* = 4). From day 4 to 11 of the experiment (i.e., 15th to 21st day of gestation), the control group had unrestricted access to food. The restricted group had access to 50% of the mean daily food intake of the control group, as it was calculated the day before. Limiting food intake to 50% is a well-known trigger for FGR in rodent experimental models (Ergaz et al., 2005). Both groups had *ad libitum* access to water.

On the 11th day (21st day of gestation) pregnant rats delivered spontaneously and the newborns were directly separated from their mother, to avoid breastfeeding, and were weighed. The newborns of the control group were used to calculate the mean birth weight and the standard deviation. Fetal growth restricted (FGR) pups were those newborns of the restricted group whose birth weight was below the mean birth weight of the control group minus two standard deviations. Those being above that cut-off, were categorized as non-FGR (Neitzke et al., 2008; Eleftheriades et al., 2014). Immediately after weighing, the newborns were matched as males or females and euthanized with inhaled sevoflurane (Sevorane, Abbott Hellas Pharmaceuticals, Greece). Brain tissues were collected directly after euthanasia, weighed and stored at −80°C.

### Quantitative Proteomics Sample Processing

A multiplex experiment was performed to include specimens from 11 male offspring (*n* = 3 control; *n* = 4 FGR; *n* = 4 non-FGR). Specimens were dissolved in 0.5 M triethylammonium bicarbonate, 0.05% sodium dodecyl sulfate and subjected to pulsed probe sonication (Misonix, Farmingdale, NY, United States). Lysates were centrifuged (16,000 g, 10 min, 4°C) and supernatants were measured for protein content using infrared spectroscopy (Merck Millipore, Darmstadt, Germany). Lysates were then reduced, alkylated and subjected to trypsin proteolysis. Peptides were labeled using the eleven-plex TMT reagent kit and analyzed using multi-dimensional liquid chromatography and tandem mass spectrometry as reported previously by the authors (Zouridis et al., 2021). As it has already been shown, three biological replicates per experimental group give sufficient statistical power to the multiplex proteomics study due to the genomic homogeneity of the animal model used (Billiard et al., 2018). Specimen selection was random and we included three pups from the control group and four from the FGR group and non-FGR group, as we expected higher phenotypic heterogeneity in the latter.

### Database Searching

Unprocessed raw files were submitted to Proteome Discoverer 1.4 for target decoy searching against the UniProtKB and TrEMBL rattus norvegicus database (release date July 2018), allowing for up to two missed cleavages, a precursor mass tolerance of 10ppm, a minimum peptide length of six and a maximum of two variable (one equal) modifications of; TMT 11-plex (Y), oxidation (M), deamidation (N, Q), or phosphorylation (S, T, Y). Methylthio (C) and TMT (K, Y and N-terminus) were set as fixed modifications. FDR at the peptide level was set at < 0.05. Percent co-isolation excluding peptides from quantitation was set at 50. Reporter ion ratios from unique peptides only were taken into consideration for the quantitation of the respective protein. The TMT ratios of proteins were median-normalized and log_2_transformed. For each experimental condition (FGR and non-FGR) the log_2_ratio over the mean of the three control mice was considered. We performed a one-sample *t*-test to identify differentially expressed proteins (DEPs) in FGR and non-FGR compared to control separately, and a two-sample *t*-test in order to identify differentially expressed proteins in FGR vs. non-FGR rats. For the two-sample *t*-test the log2ratios of proteins in FGR/control and non-FGR/control were used. The two-stage step-up method of Benjamini, Krieger and Yekutieli was used for multiple hypothesis correction (*q* < 0.1). All mass spectrometry proteomics data have been deposited to the ProteomeXchange Consortium via the PRIDE partner repository with the dataset identifier PXD011394.

### Bioinformatics Analysis

Ingenuity Pathway Analysis (IPA) (Qiagen, Hilden, Germany) was applied to differentially expressed proteins in order to identify over-represented processes in FGR and non-FGR vs. control. *P* ≤ 0.05 were considered significant.

## Results

### Animal Model

Our study’s sample consists of 111 newborn pups, divided into two groups namely the food restricted group and the control group (food restricted group vs. control group; *n* = 65, 58.6% vs. *n* = 46, 41.4%). 57 (51.4%) offspring were male (22 in the control group and 35 in the food restricted group) and 54 (48.6%) were female (24 and 30, respectively).

The mean birth weight of the control group was 6.419 g, standard deviation 0.436 g and the cut-off for FGR was set at 5.547 g as described previously. The mean birth weight of the restricted group was 5.423 g and differed significantly compared to controls (food restricted group vs. control group; 5.423 ± 0.610 g vs. 6.419 ± 0.436 g; *p* < 0.001). Moreover, the newborns of the food restricted group were divided into two further groups, namely FGR and non-FGR according to their birth weight. Those under the threshold of 5.547 g were FGR and those above that, non-FGR. Following this categorization, the restricted group consisted of 34 FGR (14 males and 20 females) and 31 non-FGR pups (21 males and 10 females). The differences in birth weight between the control group and both FGR and non-FGR were statistically significant (*p* < 0.001). Furthermore, there was statistically significant difference in birth weights between FGR and non-FGR offspring (FGR vs. non-FGR; 4.796 ± 0.479 g vs. 5.914 ± 0.479 g; p < 0.001).

Prenatal food restriction showed a remarkable sex differentiation impact on birth weight. Although male pups were heavier at birth compared to females in both control group (control males vs. control females; 6.659 ± 0.324 g vs. 6.200 ± 0.413 g; p < 0.001) and the non-FGR group (non-FGR males vs. non-FGR females; 5.930 ± 0.298 g vs. 5.880 ± 0.131 g; *p* = 0.519), FGR male newborns weighed 8.5% less than the female ones (FGR males vs. FGR females; 4.739 ± 0.629 g vs. 5.142 ± 0.240 g; *p* < 0.05). Following this observation and in order to avoid biases based on sex differentiation we decided to include only male offspring for the quantitative proteomic analysis.

Regarding brain tissue weight, there is a positive linear correlation between birth weight and brain weight (Pearson correlation coefficient = 0.295; *p* < 0.01). The food restricted group had a significantly lower brain weight compared to controls (food restricted group vs. control group; 0.150 ± 0.045 g vs. 0.180 ± 0.044 g; *p* = 0.001). The same does not apply in the food restricted group, where FGR pups have a slightly and not significantly higher brain weight than non-FGR (FGR vs. non-FGR; 0.152 ± 0.048 vs. 0.148 ± 0.043; *p* = 0.783). Table 1 summarizes body and brain weights at birth and their correlations in all studied groups.

**TABLE 1:** Birth and brain weights of the newborn pups in control, food restricted group and both subcategories of restricted group.

		Control group	Food restricted group	FGR	Non-FGR
Birth weight					
	Male	6.659 ± 0.324	5.454 ± 0.744*	4.739 ± 0.629*	5.930 ± 0.298*
	Female	6.200 ± 0.413	5.388 ± 0.410*	5.142 ± 0.240*	5.880 ± 0.131**
	Both	6.419 ± 0.436	5.423 ± 0.610*	4.976 ± 0.479*	5.914 ± 0.255*
Brain weight					
	Male	0.187 ± 0.044	0.153 ± 0.045**	0.155 ± 0.042	0.151 ± 0.048***
	Female	0.174 ± 0.044	0.147 ± 0.046***	0.149 ± 0.053	0.144 ± 0.034***
	Both	0.180 ± 0.044	0.150 ± 0.045*	0.152 ± 0.048***	0.148 ± 0.043**

*p < 0.001, **p < 0.01, and ***p < 0.05 (each value is compared with the control group). All values (mean ± standard deviation) are in grams.

### Quantitative Proteomic Analysis

**FIGURE 2:**
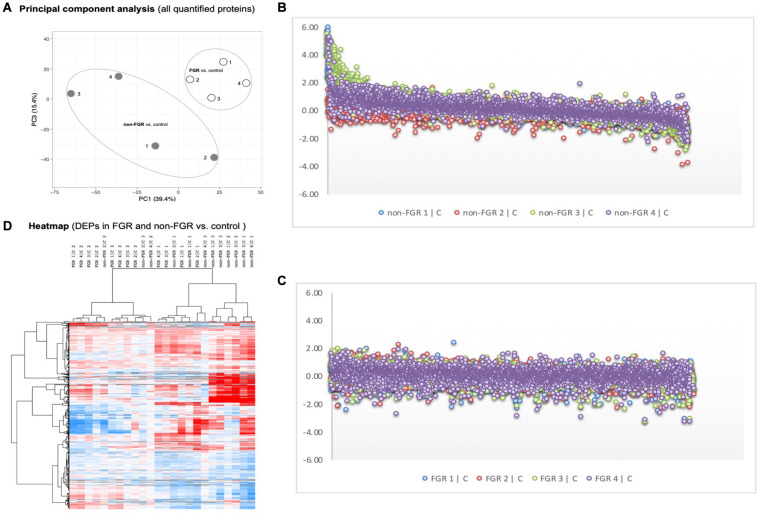
**(A)** Principal component analyses of all quantified proteins showed a distinct brain proteomic profile for FGR compared to non-FGR relative to control. **(B)** Standard deviation (SD) across the mean log2ratios of FGR vs. control. **(C)** Standard deviation (SD) across the mean log2ratios of non-FGR vs. control. **(D)** A heatmap of proteins that were differentially in FGR vs. control or non-FGR vs. control.

**FIGURE 3:**
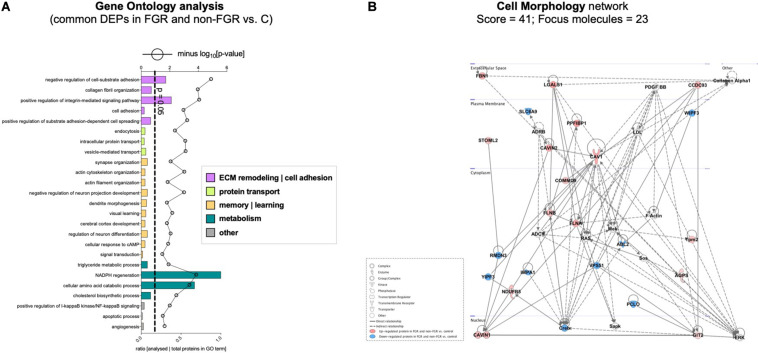
**(A)** Gene ontology analysis of the common differentially expressed proteins using DAVID showed significant enrichment for terms related to extracellular matrix (ECM) remodeling, cell adhesion, protein transport, learning, memory, metabolism and other processes, including NF-kappaB signaling, apoptosis and angiogenesis. **(B)** IPA showed an over-representation of a direct protein interaction network associated with cell morphology in both FGR and non-FGR groups vs. control.

**FIGURE 4:**
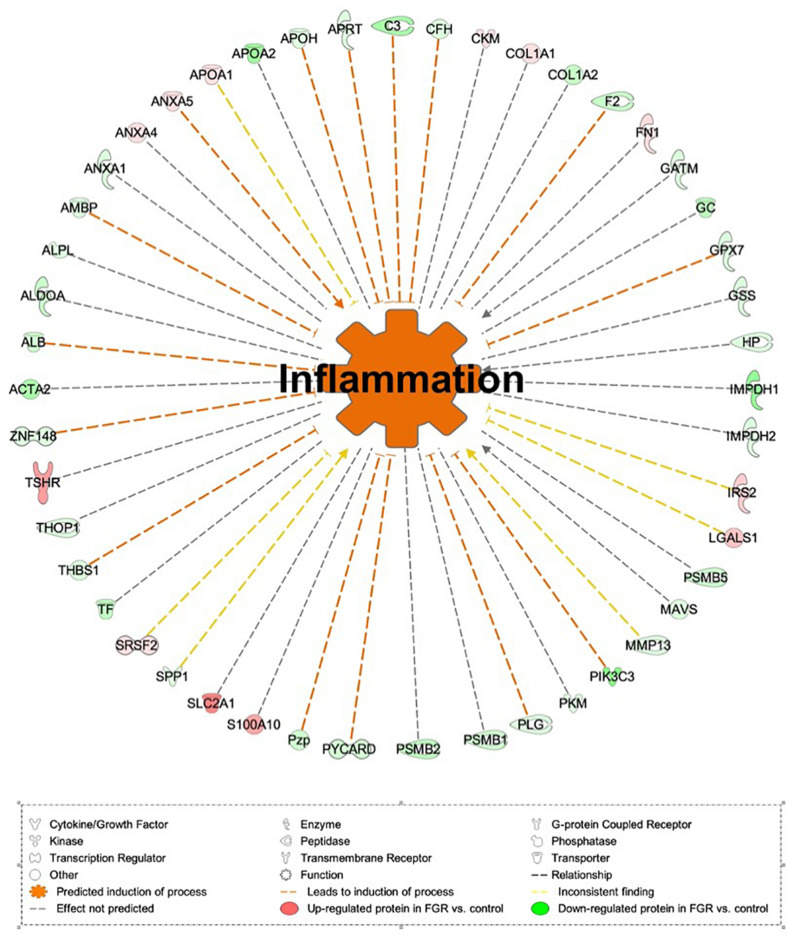
IPA showed significant induction of inflammation in FGR pups.

**FIGURE 5:**
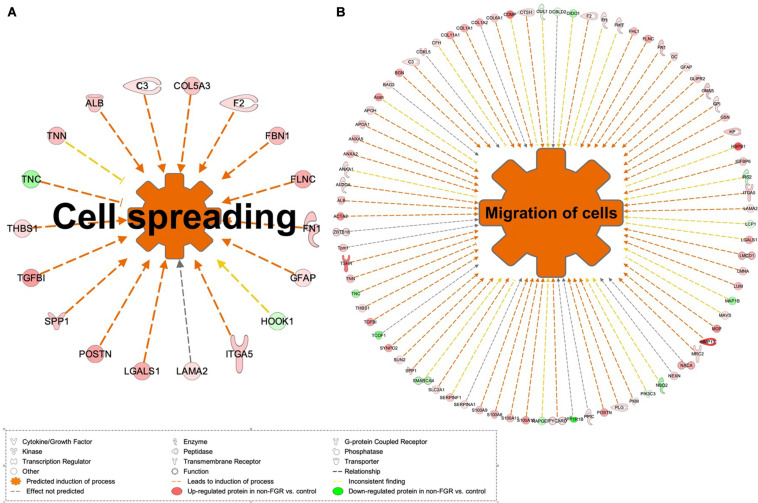
**(A)** IPA showed significant induction of cell migration and **(B)** cell spreading in non-FGR rats.

Proteomic analysis resulted in the profiling of 3,964 proteins (peptide FDR, *p* < 0.05) (Supplementary Table 1). Principal component analyses of all quantified proteins showed a distinct brain proteomic profile for FGR compared to non-FGR pups relative to control (Figure 2A). The standard deviation (SD) across the mean log_2_ratios of FGR vs. control and non-FGR vs. control were 0.39 and 0.61, respectively (Figures 2B,C). Of the quantified proteins, 766 were differentially expressed in FGR vs. control and 648 in non-FGR vs. control (Supplementary Tables 2, 3). A heatmap of proteins that were differentially in FGR vs. control or non-FGR vs. control are illustrated in heatmap format in Figure 2D. Of these, 235 proteins were commonly up- or down-regulated in both FGR and non-FGR vs. controls (Supplementary Table 4) and 391 were differentially expressed in FGR vs. non-FGR. Gene ontology analysis of the common differentially expressed proteins using DAVID showed significant enrichment for terms related to extracellular matrix (ECM) remodeling, cell adhesion, protein transport, learning, memory, metabolism and other processes, including NF-kappaB signaling, apoptosis and angiogenesis (Figure 3A). IPA showed an over-representation of a direct protein interaction network associated with cell morphology in both FGR and non-FGR groups vs. control (Figure 3B). IPA also showed significant induction of inflammation in FGR pups (z-score = 2.3; *p* = 4.5e-6) (Figure 4), and significant induction of cell migration (z-score = 3.9; *p* = 2.6e-5) and cell spreading (z-score = 3.2; *p* = 2.9e-5) in non-FGR rats (Figure 5).

## Discussion

Our study investigated the differences in global brain proteome between growth restricted offspring and those that experienced the same adverse prenatal environment without developing growth restriction. The aim was thus to identify the impact of maternal malnutrition on brain proteomic profile of the offspring and the possible contribution of birth weight *per se* on unfavorable outcomes. Hence, we investigated the common differentially expressed proteins in these two aforementioned groups (FGR and non-FGR) vs. control and the differentially expressed proteins of each group vs. control. Eventually, we investigated protein expression pathways that are either common or unique in FGR and non-FGR groups.

### Common Differentially Expressed Proteins in Food Restricted Offspring (FGR and Non-FGR) vs. Control

#### IPA Showed an Over-Representation of a Direct Protein Interaction Network Associated With Cell Morphology

In our study, bioinformatics analysis showed over-representation of a direct protein interaction network regarding Cell Morphology (Score = 41, *n* = 23 proteins) in FGR and non-FGR vs. control, with CAVIN1 (Caveolae-associated protein 1), CAVIN2 (Caveolae-associated protein 2), CAV1 (Caveolin-1), FLNA (Filamin-A) and FLNB (Filamin-B) as key components to that network (Figure 3B). CAVIN1, CAVIN2 and CAV1 are associated with caveolae formation and organization. Caveolae are plasma membrane invaginations containing cholesterol and sphingolipids. Their role is critical in endocytosis, lipid transportation and regulation of signal transduction (Parton and del Pozo, 2013). The formation and function of caveolae require the interconnected presence of both Caveolins and Cavins (Hansen and Nichols, 2010). Interestingly, CAV1 has an impact on the initiation of neuritogenesis, mainly as part of the membrane/lipid rafts (MLRs), which regulate the pro-growth signaling events (Kamiguchi, 2006). MLRs are important for neurite growth and guidance as well as synapse formation (Willmann et al., 2006; Guirland and Zheng, 2007). CAV1 expression in corticohippocampal neurons can increase pro-growth signaling and promote dendritic growth and branching of primary neurons (Head et al., 2011). On the other hand, loss of CAV1 can lead to disruption of MLRs and inhibition of neuritogenesis (Nakai and Kamiguchi, 2002). Regarding Filamins, it is known that Filamin-A promotes branching of actin filaments, anchors transmembrane proteins to the actin cytoskeleton and also serves as a scaffold for cytoplasmic signaling proteins. In interaction with Filamin-B, Filamin-A allows neuroblast migration from the ventricular zone into the cortical plate (Sheen et al., 2002). FLNA is also involved in ciliogenesis and has an important role in cell-to-cell contacts and junctions during brain development (Adams et al., 2012). At least 10 different regulators of FLNA are able to regulate neuronal migration, revealing its fundamental role in brain development (Sarkisian et al., 2008). Furthermore, in animal studies maternal caloric restriction is related to actin-related proteins and can disturb cell multiplication and impair cell differentiation (Fletcher and Mullins, 2010). These findings stress that cytoskeleton formation during development may provide a platform to overcome *in utero* effects on postnatal life (Lee et al., 2018).

#### Significant Enrichment for Terms Related to Positive Regulation of NF-KappaB Signaling

Furthermore, in our study we found significant enrichment for terms related to positive regulation of I-kappaB kinase and NF-kappaB signaling (Figure 3A). NF-kappaB signaling includes all the processes in which a signal activates the I-kappaB-kinase (IKK) complex and leads to NF-kappaB dimers, which translocate to the nucleus and regulate transcription (Santoro et al., 2003). The transcription factor NF-kappaB is regulated during the development of the central nervous system but also during inflammation and brain injury (Mattson et al., 2000). Thus, it has a dual role, both as a protective factor and as an activator of apoptosis pathways (O’Neill and Kaltschmidt, 1997). Genetic silencing of the NF-kappaB pathway in mice temporal lobe can result in neuronal degeneration and impeded axogenesis and synaptogenesis (Imielski et al., 2012). It is also observed that expression of NF-kappa B in murine hippocampus has a regulatory effect on excitatory synapse and dendritic morphology not only in a developmental but also in a mature stage (Boersma et al., 2011). Schmeisser et al. (2012), report that inhibition of I-kappaB-kinase (IKK) can lead to reduced basal synaptic transmission and spatial learning. On the other hand, activation of IKK is able to induce synaptic adaptation and behavioral adjustments (Christoffel et al., 2011). Moreover, except for its regulatory role in neural development, NF-kappaB can also affect learning and memory functions (Gutierrez and Davies, 2011; Caroni et al., 2012).

#### Significant Enrichment for Terms Related to Memory and Learning Processes

Regarding memory and learning processes, our study showed significant enrichment of actin filament and cytoskeleton organization, negative regulation of neuron projection, dendrite morphogenesis, cerebral cortex development, neuron differentiation and visual learning (Figure 3A). In growth restriction, hippocampus, cerebellum and neocortex undergo not only structural but also functional alterations and have been linked to poor spatial memory, lower performance in school, and lower intelligence quotient in humans (Leitner et al., 2005, 2007) as well as in animal models (Caprau et al., 2012; Fung et al., 2012). A recent whole brain connectome study in FGR children by Batalle et al. (2012), revealed reduced network efficiency and connectivity in the prefrontal and limbic networks. These brain observations also correlate with hyperactivity or cognitive deficits at school age (Fischi-Gomez et al., 2015). Interestingly, we found that Tyrosine-protein kinase (Abl2) was down-regulated in both FGR and non-FGR (Supplementary Table 4). Tyrosine-protein kinase (Abl2) is a non-receptor tyrosine-protein kinase that regulates processes related to cell growth and survival such as actin and cytoskeleton remodeling in response to extracellular stimuli, autophagy, DNA damage response and apoptosis (Wang et al., 2001; Hu et al., 2005). Via its adhesion-dependent phosphorylation of Rho GTPase-activating protein (GAP) which inhibits Rho GAP activity, it acts as a dendrite stabilization factor (Shapiro et al., 2017). In previous rodent models, chronic stress led to decreased levels of Tyrosine-protein kinase (Abl2) and was associated with dendritic structure disruption and atrophy (McEwen et al., 2016; Conrad et al., 2017). Similarly, in our study, food restricted pups displayed a structural response similar to a chronic stressor exposure, irrespective of birth weight status.

### Common Differentially Expressed Proteins in FGR vs. Control Showed Significant Induction of Inflammation Process

Our study showed significant induction of inflammation process (z-score = 2.3; *p* = 4.5e-6) in FGR rats vs. control. Complement C3 (C3), Complement factor H (CFH) and Annexin A5 (ANXA5) are key components toward the induction of the process. Inflammation following traumatic brain injury (TBI) incidents has been extensively studied and is thought to be a major component of the degenerative events that follow brain injuries (Blennow et al., 2012). The complement system contributes to inflammation and promotes neuronal loss, edema and inflammatory cellular infiltration (Ruseva et al., 2015; Rich et al., 2016). In an experimental mice model, complement 3 inhibition locally improved acute and subacute outcomes and its inhibition is required to overcome chronic inflammation and progressive neuronal loss (Alawieh and Tomlinson, 2016; Alawieh et al., 2018). On the other hand, activation of C3 leads to microglial and astrocyte activation and reduced dendritic and synaptic density (Alawieh et al., 2018). Regarding Complement factor H (CFH), it is a glycoprotein that acts as a soluble inhibitor of the alternative complement pathway by preventing the local formation of C3b in the complement amplification loop (Wu et al., 2009; Blaum et al., 2015). In our study, both C3 and CFH were down-regulated in FGR group vs. control with predicted action the induction of inflammation. Interestingly, in the non-FGR vs. control both C3 and CFH were up-regulated. Furthermore, Annexin A5 (ANXA5) is known to have anti-inflammatory and anti-apoptotic properties and at the same time, it may serve as a diagnostic tool in visualizing cell death (Boersma et al., 2005). The aforementioned effects of ANXA5 were demonstrated in a rabbit experimental model, in which diannexin, a dimer of ANXA5, was used as a treatment after myocardial ischemia and reperfusion with cardioprotective abilities by reducing no-reflow areas (Hale et al., 2011). The same results were also shown in a mice model, were ANXA5 treatment was associated with reduced post-infract inflammatory response and improved cardiac function (de Jong et al., 2018). In our study, ANXA5 was up-regulated indicating a potential anti-inflammatory neuro-protective mechanism similar to that after a myocardial infract.

### Common Differentially Expressed Proteins in Non-FGR vs. Control Showed Significant Induction of Cell Migration and Cell Spreading

Our study showed that cell migration (z-score = 3.9; *p* = 2.6e-5) and cell spreading (z-score = 3.2; *p* = 2.9e-5) were induced in non-FGR rats (Figure 5). Key components of these processes were matrix metalloproteinase 13 (MMP13, also known as Collagenase-3), protein S100A family and glial fibrillary acidic protein (GFAP). Interestingly, in our study, MMP13 was strongly up-regulated in the non-FGR group vs. control and down-regulated in the FGR group. MMP13 plays a significant role in the degradation of extracellular matrix proteins including fibrillar collagen, fibronectin, TNC and ACAN, cleaves collagen II and indicates the highest gelatinase activity among the collagenases (Knauper et al., 1996). In a recent experimental rodent model, it was revealed that MMP13 has a central position in the activation of other MMPs and orchestrates neurovascular remodeling in case of brain infract. Silencing of MMP13 resulted in a reduced amount of new neuroblasts in the infract areas (Ma et al., 2016). Our findings, that is, up-regulation of MMP13, ACAN, and FN1 and down-regulation of TNC, suggest that a neuroprotective and neurorepair mechanism was initiated in the non-FGR pups, which was similar to a cerebral post-ischemic mechanism. S100A9 is a calcium- and zinc-binding protein which plays a significant role in the regulation of inflammatory processes and immune response by inducing chemotaxis, cell death and apoptosis. The complex S100A9/S100A8 is also known as calprotectin and has a wide variety of intra- and extracellular functions (Koike et al., 2012). Both have been associated with neuro-inflammatory and neuro-degenerative diseases as well as poor cognitive and motor development in infants (Haque et al., 2018). Hence, our results indicate that non-FGR pups are under an inflammatory state.

Regarding GFAP, an astrocyte signature protein, we found a significant up-regulation in the non-FGR group vs. control, whereas GFAP was down-regulated in the FGR group. Astrocytes development starts at late embryonic life and increases in the first month postnatally. In rodents, this process is amplified in the first postnatal week, when astrocytes gain increased cytoskeletal complexity and form overlapping networks (Catalani et al., 2002). GFAP expression is a late benchmark in astrocyte development and maturation and our research outcomes stress that food restriction can lead to a precocious differentiation of astrocytes strengthening the hypothesis of astrocytes as possible drivers of neurodevelopmental disorders. Similar results have been published in a low-protein intake rodent model and underline that developing brain is highly susceptible to *in utero* insults (Naik et al., 2017). Moreover, GFAP up-regulation can induce a reactive state of astrocytes, characterized by cellular hypertrophy, known as reactive gliosis or astrogliosis, which in severe cases can result in increased proliferation and glial scar formation (Hol and Pekny, 2015). It is already known that astrocytes are a key element in brain function, able to receive and integrate a number of signals from the endogenous and exogenous environment, modify neuronal function with an array of processes including myelination, energy metabolism, synaptic development as well as dendritic maturation and integration and thus, any late embryonic disturbing event can have severe, acute and permanent implications (Abbink et al., 2019).

## Conclusion

To our knowledge, the present study constitutes the most systematic and comprehensive whole-brain proteomic analysis to date providing important insights into the effects of prenatal food restricted environment in FGR and non-FGR offspring. Our study demonstrated that a wide range of proteomic alterations take place in the brain of both FGR and non-FGR pups as a result of poor maternal nutrition. These changes are indicative of an increased initiation of neuritogenesis, neurite growth, synapse formation and dendritic branching of primary neurons. Although these processes may provide an adaptive mechanism to prenatal adversities, they lead to a response similar to chronic stress that has been linked to poor spatial memory, lower performance at school, and lower intelligence quotient in humans. Regarding FGR offspring, our study showed that inflammation process is induced, and the pups are under a chronic inflammatory state. Furthermore, our experimental study showed that in non-FGR offspring cell migration and cell spreading processes are induced. The induction of the aforementioned processes implicates that a neuroprotective and neurorepair mechanism was initiated in the non-FGR pups. In conclusion, this study underlines the distinct proteomic profile of FGR and non-FGR offspring of food restricted Wistar dams, showing the importance of both prenatal environment and birth weight in brain development. Ultimately, this study highlighted that not only FGR, but also appropriately grown pups, which have been exposed to prenatal food deprivation may be at increased risk for impaired cognitive and developmental outcomes.

### Study Limitation

Due to the experimental nature of our study extrapolating the results to humans should be made with caution, as in every animal study. Parameters such as the use of certain animal model, its variety of developmental or metabolic pathways and the number of animals in each experimental group that should be kept to the minimum may limit the strength of our results.

## Data Availability Statement

The datasets presented in this study have been deposited to the ProteomeXchange Consortium via the PRIDE partner repository with the dataset identifier PXD011394, http://proteomecentral.proteomexchange.org/cgi/GetDataset?ID=PXD011394.

## Ethics Statement

The animal study was reviewed and approved by Ethics Committee of Aretaieion University Hospital, Medical School of the National and Kapodistrian University of Athens with registration number B-207/13-10-2016. Research license and approval for experimental animal (RjHan:WI–Wistar rats) utilization was granted by the Division of Agriculture and Veterinary Policy, District of Attica, Greece (Decision 5035/21-09-2017 and its modification 1211/19-03-2018).

## Author Contributions

SG and ME jointly led the study and supervised the research. AP, AZ, P-MS, and ME designed and performed the research. AM and SG analyzed the data and performed the bioinformatics analysis. GE, DP, and ED provided feedback regarding the research design and manuscript editing. AP, AM, PP, and ME wrote the manuscript with input from all co-authors. All authors contributed to the article and approved the submitted version.

## Conflict of Interest

SG was Founder, President and CEO of Proteas Bioanalytics Inc., BioLabs at the Lundquist Institute. The authors declare that this study received funding from Procter & Gamble Hellas. The funder was not involved in the study design, collection, analysis, interpretation of data, the writing of this article or the decision to submit it for publication.

## Supplementary Material

Supplementary Table 1 All identified proteins (peptide FDR *p* < 0.05) by the proteomic analysis.

Supplementary Table 2 Differentially expressed proteins (DEPs) in FGR vs. control.

Supplementary Table 3 Differentially expressed proteins (DEPs) in non-FGR vs. control.

Supplementary Table 4 Commonly up- or down-regulated proteins in both FGR and non-FGR vs. controls.
